# Effect of X-Irradiation at Different Stages in the Cell Cycle on Individual Cell–Based Kinetics in an Asynchronous Cell Population

**DOI:** 10.1371/journal.pone.0128090

**Published:** 2015-06-18

**Authors:** Eri Tsuchida, Atsushi Kaida, Endrawan Pratama, Masa-Aki Ikeda, Keiji Suzuki, Kiyoshi Harada, Masahiko Miura

**Affiliations:** 1 Section of Oral Radiation Oncology, Department of Oral Health Sciences, Graduate School of Medical and Dental Sciences, Tokyo Medical and Dental University, 1-5-45 Yushima, Bunkyo-ku, Tokyo, 113–8549, Japan; 2 Section of Maxillofacial Surgery, Department of Maxillofacial and Neck Reconstruction, Graduate School of Medical and Dental Sciences, Tokyo Medical and Dental University, 1-5-45 Yushima, Bunkyo-ku, Tokyo, 113–8549, Japan; 3 Section of Molecular Craniofacial Embryology, Graduate School of Medical and Dental Sciences, Tokyo Medical and Dental University, 1-5-45 Yushima, Bunkyo-ku, Tokyo, 113–8549, Japan; 4 Department of Radiation Medical Sciences, Atomic Bomb Disease Institute, Nagasaki University Graduate School of Biomedical Sciences, 1-12-4 Sakamoto, Nagasaki, 852–8523, Japan; ENEA, ITALY

## Abstract

Using an asynchronously growing cell population, we investigated how X-irradiation at different stages of the cell cycle influences individual cell–based kinetics. To visualize the cell-cycle phase, we employed the fluorescent ubiquitination-based cell cycle indicator (Fucci). After 5 Gy irradiation, HeLa cells no longer entered M phase in an order determined by their previous stage of the cell cycle, primarily because green phase (S and G2) was less prolonged in cells irradiated during the red phase (G1) than in those irradiated during the green phase. Furthermore, prolongation of the green phase in cells irradiated during the red phase gradually increased as the irradiation timing approached late G1 phase. The results revealed that endoreduplication rarely occurs in this cell line under the conditions we studied. We next established a method for classifying the green phase into early S, mid S, late S, and G2 phases at the time of irradiation, and then attempted to estimate the duration of G2 arrest based on certain assumptions. The value was the largest when cells were irradiated in mid or late S phase and the smallest when they were irradiated in G1 phase. In this study, by closely following individual cells irradiated at different cell-cycle phases, we revealed for the first time the unique cell-cycle kinetics in HeLa cells that follow irradiation.

## Introduction

The study of cell-cycle kinetics essentially started with the development of autoradiography using ^3^H-labeled thymidine [1]; subsequently, the percent-labeled mitosis technique accelerated the progress of the field [2]. ^3^H-labeled thymidine was then replaced by bromodeoxyuridine (BrdU), which is detected by immunostaining with an anti-BrdU antibody, and the speed of analysis was improved by the emergence of flow cytometry [3, 4]. As these methodologies developed, they were used to study the effects of ionizing radiation on cell cycle kinetics [5]. In combination with the concept of cell-cycle checkpoints [6], the kinetics of the distinct G2 arrest that occurs in p53-defective tumor cells have been extensively studied [7, 8]. Recent studies have elucidated the molecular mechanisms associated with the ATR/Chk1 and ATM/Chk2 pathways, which are potential targets for radiosensitizing agents [9]. DNA repair is thought to occur efficiently during G2 arrest by halting cell-cycle progression; indeed, radioresistance and the duration of G2 arrest are positively correlated [10]. On the other hand, radiosensitization after poly ADP-ribose polymerase (PARP) inhibition is accompanied by elongation of G2 arrest [11]. Therefore, it is possible that inefficient DNA repair prolongs G2 arrest, leading to increased cellular radiosensitivity. Consequently, the duration of G2 arrest should be carefully considered in the discussions of correlates of radiosensitivity.

In most studies, the proportion of cells in G2/M phase, based on DNA content in the whole population following irradiation, has been determined by flow-cytometric analysis [12]. However, this approach is unable to reveal how cells irradiated in each phase of the cell cycle contribute separately to G2 arrest. In order to examine such effects, it is necessary to isolate a synchronized population. Terasima and Tolmach were the first to successfully collect mitotic cells by the shake-off method, and their study revealed that radiosensitivity changes dramatically as a synchronized cell population progresses through the cell cycle [13]. Similarly, in synchronously growing cell populations originating from collected mitotic cells, growth delay is also strongly dependent on the cell-cycle phase at which cells were irradiated [14]. Various drugs, including hydroxyurea, lovastatin, thymidine, and nocodazole, which halt cell-cycle progression at specific phases, have also been used to generate synchronous cell populations [15]. However, imperfections in synchronization, redistribution after release of synchronization, and the side effects of drugs pose technical challenges to the interpretation of these experiments; for instance, hydroxyurea induces massive amounts of DNA double-strand breaks (DSBs) [16]. Furthermore, when cells are simultaneously irradiated under asynchronous conditions, independent analysis of each separate population makes it difficult to compare and reconstruct cell-cycle kinetics. Therefore, cell-cycle markers that can be visualized in living cells, in combination with time-lapse imaging, would allow us to overcome such issues and obtain more precise information.

In addition to cell-cycle checkpoints, endoreduplication occurs in p53-deficient cancer cells after exposure to high doses of ionizing radiation[17–20] or etoposide [21]: specifically, cells skip mitosis after irradiation, resulting in multiple rounds of DNA replication and chromosome segregation without cytokinesis, giving rise to endopolyploid giant cells [17, 18]. p21 is transcriptionally activated by p53 after irradiation, and is thought to play a pivotal role in inhibiting endoreduplication [17]. However, cells with functional p53 are also likely to exhibit endoreduplication following exposure to DNA-damaging agents, including irradiation and etoposide, depending on the extent of the damage [19, 20]. Therefore, irrespective of p53 status, we must carefully observe whether endoreduplication occurs in order to precisely interpret radiation-induced cell-cycle kinetics.

Levels of the Cdt1 protein, a DNA replication licensing factor, are regulated along with the cell cycle by the E3 ligase SCF^Skp2^, enabling specific expression of Cdt1 in G1 phase [22, 23]. On the other hand, Geminin is an inhibitor of Cdt1 whose levels are regulated by another E3 ligase, APC^Cdh1^, enabling expression of this protein in the S/G2/M phases [23]. Taking advantage of these properties, Sakaue-Sawano et al. developed the fluorescent ubiquitination-based cell cycle indicator (Fucci): cells expressing the Fucci probes emit red fluorescence in G1 and green fluorescence in the other phases [24]. We employed this technique and succeeded in visualizing radiation-induced G2 arrest kinetics in HeLa cells expressing the Fucci probes [25, 26].

In this study, we attempted to closely monitor individual cells irradiated at different cell-cycle phases in an asynchronous population, without isolating synchronized cell populations. The Fucci system was able to discriminate G2-arrested cells from cells undergoing mis-segregation during the endoreduplication process: specifically, in the latter case, the color of the cell turns from green to red without cytokinesis [19, 21], allowing mis-segregated cells to be captured by time-lapse imaging. Our results reveal, for the first time, the unique cell-cycle kinetics that follow irradiation.

## Materials and Methods

### Cell lines and culture conditions

HeLa cells expressing the Fucci probes (HeLa-Fucci) were provided by the RIKEN BRC through the National Bio-Resource Project of MEXT, Japan. In the Fucci system, cells emitting red, green, or orange (red plus green) fluorescence correspond to G1, S/G2/M, and early S phase, respectively [24]. BJ1-hTERT-Fucci cells expressing the Fucci probes were established from h-TERT-immortalized normal human diploid foreskin fibroblasts (BJ-hTERT) [27] using a previously described method [19]. All cell lines were maintained at 37°C in a 5% CO_2_ humidified atmosphere in DMEM (Sigma-Aldrich, St. Louis, MO) supplemented with 10% fetal bovine serum. To express wild-type p53 in HeLa-Fucci cells, cells were infected with an adenovirus expressing wild-type p53 (Ad-p53) as described previously [28]. Percentage of labeled mitoses [2] was determined by measuring the proportion of labeled mitotic cells at the indicated times after incorporation of 10 μM 5-ethynyl-2’-deoxyuridine (EdU) for 20 min, using the Click-iT EdU Alexa Fluor 647 Imaging Kit (Invitrogen, Carlsbad, CA), rather than ^3^H-labeled thymidine.

### Time-lapse Imaging

Cells were imaged with a BIOREVO BZ-9000 fluorescence microscope (KEYENCE, Osaka, Japan). Images were acquired every 60 or 120 min. During imaging, cells were held in an incubation chamber at 37°C in a humidified atmosphere containing 95% air / 5% CO_2_ (Tokai Hit, Fujinomiya, Japan). After irradiation, each cell was monitored until the next mitosis, and the changes in fluorescent colors and their durations were recorded. Green fluorescence intensity within each nucleus was quantitated using the Dynamic Cell Count software (KEYENCE). To adjust the fluorescence intensities among different fields, the background fluorescence intensity of each image was normalized to 1. A common database obtained from 627 cells flash-labeled with EdU immediately after 5 Gy irradiation was obtained for the analysis.

### Flow-cytometric analysis

For DNA-content analysis, trypsinized cells were centrifuged, and the pellets were washed in PBS. Cells were fixed in 4% paraformaldehyde in PBS for 15 min on ice and washed in PBS. Cells were then stained with Hoechst 33342 for 30 min. For analysis of labeled mitosis, the Click-iT EdU Pacific Blue Flow Cytometry Assay kit (Invitrogen) was used in combination with an M-phase marker, phospho-histone H3. Briefly, cells were incubated in growth medium containing 10 μM EdU (Invitrogen) for 20 min immediately after irradiation, and then washed in PBS. After the indicated time, cells were fixed in 4% paraformaldehyde in PBS for 15 min, and then stained using azide conjugated with Pacific Blue. Next, cells were incubated for 1 h on ice with primary monoclonal antibody against phospho-histone H3 (Ser10) (1:50; Cell Signaling, Danvers, MA). Following extensive washing, cells were incubated in goat anti-mouse IgG secondary antibody conjugated to Alexa Fluor 647 (1:500; Invitrogen) for 30 min on ice. After staining, all samples were washed in PBS. Finally, single-cell suspensions were passed through a nylon mesh. Each sample was analyzed on a FACS CantoII cytometer (Becton Dickinson, Franklin Lakes, NJ) using the FlowJo software (Tree Star, Ashland, OR, USA). At least 10^3^ mitotic cells were analyzed for each time point. Duration of S phase was defined as the interval between 50% levels in the ascending and descending portions of the curve. Duration of G2 plus 1/2 M was determined from the interval between the 0% and 50% level on the ascending portion of the curve.

### Irradiation

Cells were irradiated at the indicated doses (in most cases, 5 Gy) with an RX-650 cabinet X-Radiator system (130 kVp, 5 mA, 0.5 mm Al filtration) (Faxitron, Tucson, AZ) at a dose rate ranging from 0.7 to 0.8 Gy/ min.

### Determination of S-phase delay following irradiation using synchronous cell populations

Cells were synchronized with a double–thymidine block method. Cells (10^5^ cells per 60 mm dish) were seeded 18–24 h prior to the first application of thymidine, and then cultured in complete medium supplemented with 2 mM thymidine for 14 h. Cells were then released from the block by changing the medium to thymidine-free complete growth medium for 9 h, and finally incubated for 14 h in medium supplemented with 2 mM thymidine. Cells were then washed in PBS, incubated in complete medium without thymidine, and collected 1 h after release.

### Fluorescence immunostaining

Twenty-four hours after Ad-p53 infection, HeLa-Fucci cells were fixed in 4% paraformaldehyde in PBS for 15 min, and then extensively washed in PBS. Cells were then incubated for 1 h with primary monoclonal antibody against p53 (DO-1) (1:500; Santa Cruz Biotechnology, Dallas, TX). After washing in PBS-T, cells were incubated in goat anti-mouse IgG secondary antibody conjugated with Alexa Fluor 647 (1:500; Invitrogen) for 30 min. Images were obtained using a FLUOVIEW FV10i (Olympus, Tokyo, Japan) and analyzed with the FV10-ASW Viewer software, version 4.0 (Olympus).

### Statistical analysis

One way ANOVA with post hoc Dunnett’s test or Mann–Whitney U test was used for statistical analysis. *P* values < 0.05 were considered statistically significant. Regression lines were obtained by the least-squares method.

## Results

### Characterization of cell kinetics in unirradiated, exponentially growing HeLa-Fucci cells

**Fig 1:**
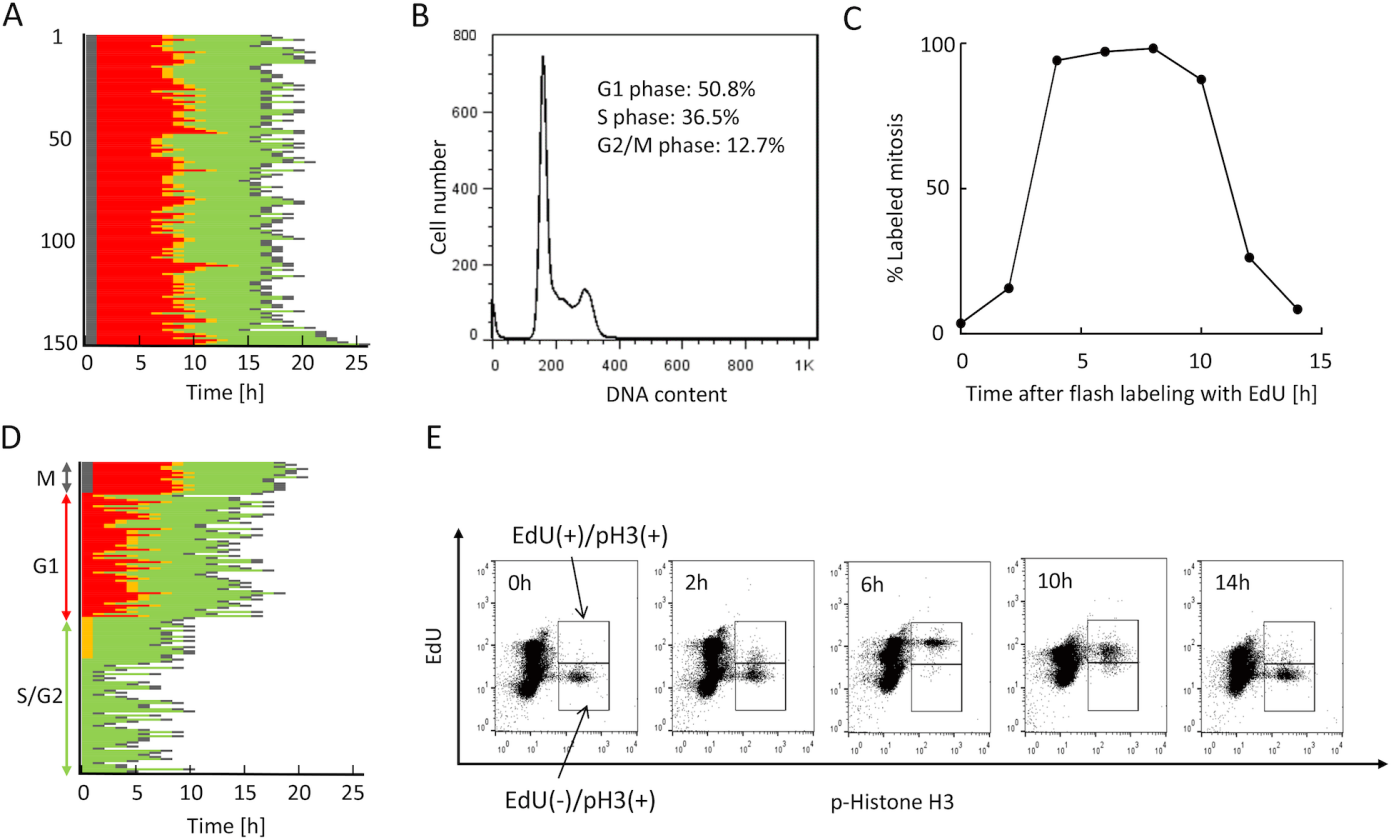
Characterization of cell-cycle kinetics in exponentially growing HeLa-Fucci cells. (A) Pedigree analysis from one M phase to the next M phase. Real-time imaging analysis was performed based on 150 HeLa-Fucci cells. Each bar represents one cell cycle, starting from one M phase to the next M phase, consisting of M (black), red, orange, and green phases. Orange represents cells in which both red and green fluorescence signals were emitted during early S phase. (B) DNA-content analysis by flow cytometry using Hoechst 33342. Proportion of each cell-cycle phase is indicated. (C) Percent-labeled mitosis index. Cells were flash-labeled with EdU, and labeled mitotic cells were detected by flow cytometry. Two-dimensional analysis (phospho-histone H3 and EdU) was performed at the indicated times after flash-EdU labeling. (D) Order of progression to M phase in red and green cells. Mitotic, red, and green cells at the start of observation were monitored until the next entry into M phase. (E) Time course of EdU-labeled mitotic cells. Representative two-dimensional flow-cytometric plots are shown at the indicated times after flash EdU-labeling described in Fig 1C.

We first determined the durations of each cell-cycle phase in exponentially growing HeLa-Fucci cells. Fig 1A shows the changes in fluorescence colors and the cell-cycle durations (from M phase to the next M phase) obtained by time-lapse imaging of 150 cells. Duration of red phase (G1), green phase (S/G2), M phase, and the whole cell cycle were calculated from the mean values from 150 cells. From flow-cytometric analysis of DNA content, the duration of each phase was determined from the corresponding proportions, assuming an exponential distribution as described by Watanabe and Okada et al. [29] (Fig 1B). Furthermore, we examined the durations of S, G2, and G1 phases by the percent-labeled mitosis technique [2, 5], using the recently developed EdU detection method instead of ^3^H-labeled thymidine or BrdU. Flow cytometry was used to detect EdU-labeled mitosis, using phospho-histone H3 as an M-phase marker (Fig 1C). Cell-cycle durations determined in three different ways are summarized in Table 1; all of these values were consistent with one another.

**Table 1:** Durations (h) of each cell-cycle phase in exponentially growing HeLa-Fucci cells.

Phase	Fucci analysis^1^ ^)^	DNA content analysis^2^ ^)^	Percent-labeled mitosis analysis^3^ ^)^
**G1**	7.1	7.2	6.5
**S**		7.0	7.3
**G2**		2.0	2.2
**S+G2**	9.0	9.0	9.5
**M**	1.0	(1.0)	(1.0)
**Total** ^**4**^ ^**)**^	17	(17)	(17)

1) From Fig 1A, G1-phase duration was determined from the red-phase duration. S+G2-phase duration was determined from the green-phase duration. Values represent means from 150 cells. Mitotic cells were identified morphologically. Total cell-cycle time was calculated from the duration between one M phase and the next M phase, as determined by time-lapse imaging.

2) Each cell-cycle phase duration was calculated from the proportion of each cell cycle phase (Fig 1B), assuming an exponential distribution according to the equations as described by Watanabe and Okada [29]. Values in parenthesis were derived from Fucci analysis.

3) Durations of S and G2 phases were directly determined from the curve in Fig 1C, as described in Materials and Methods. G1-phase duration was determined by subtracting the S+G2+M duration from the total cell-cycle time.

4) The actual culture doubling time obtained from growth curves was 17–18 h.

Taking advantage of the properties of Fucci, we next analyzed the order of entry of red- and green-phase cell populations into M phase. Sixty red cells and ninety green cells at the start of observation were sorted separately, as shown in Fig 1D. This result clearly shows that green cells entered M phase first, whereas red cells entered M phase after almost all the green cells had done so. Fig 1E shows representative plots at the indicated times after the EdU labeling performed for Fig 1C. EdU-labeled mitotic cells started to appear around 2 h after labeling, corresponding to the duration of G2 phase, reaching ~100% around 6 h after labeling. Subsequently, the percentage decreased to just over 0% for up to 14 h (Fig 1E). The G2, S, and G1 fractions (EdU-negative, EdU-positive, and EdU-negative, respectively) entered M phase in that order, in keeping with the observations shown in Fig 1D.

### Effects of irradiation dose on the prolongation of red or green phase

**Fig 2:**
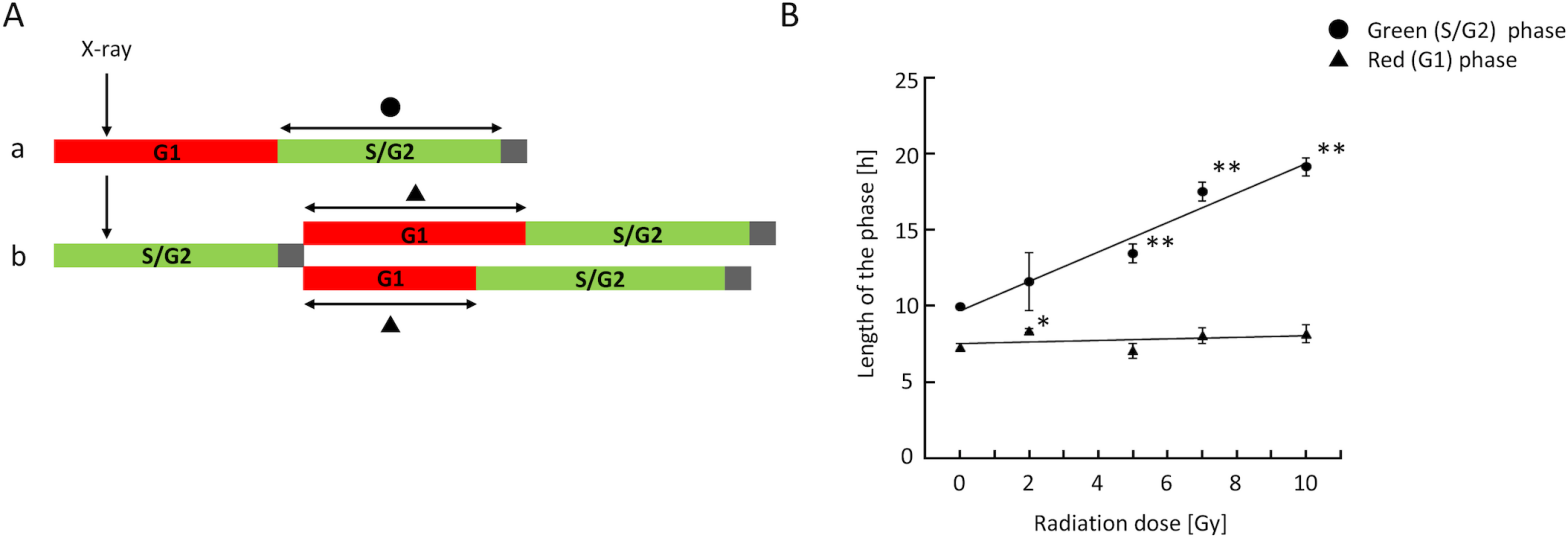
Dose-dependency of prolongation of red and green phase after irradiation. (A) Schematic presentation of irradiation timing and measurement of the duration of green (a) or red (b) phase. Black square represents M phase. In panel b, the two daughter cells did not exhibit exactly the same red-phase duration; therefore, each value was separately measured and mean values were calculated. (B) Dose-dependency of prolongation of red and green phases after irradiation. Data represent means ± S.D. of values obtained from at least four different fields for each dose. Each field contained at least 10 cells. *, p < 0.05; **, p < 0.01 vs. control values at 0 Gy (one way ANOVA with post hoc Dunnett’s test).

To obtain individual cell–based information regarding prolongation of the red and green phases, we first set up simple experimental sequences, as depicted in Fig 2A. We examined how long the green phase was prolonged when cells in red phase were irradiated, and *vice versa*. The dose dependency is shown in Fig 2B. When cells in red phase were irradiated, the duration of the green phase excluding M phase (black square) increased in a dose-dependent manner. On the other hand, when cells in green phase were irradiated at doses up to 10 Gy, the duration of red phase did not significantly increase.

### Following irradiation, cells no longer enter M phase in order of cell-cycle phase

**Fig 3:**
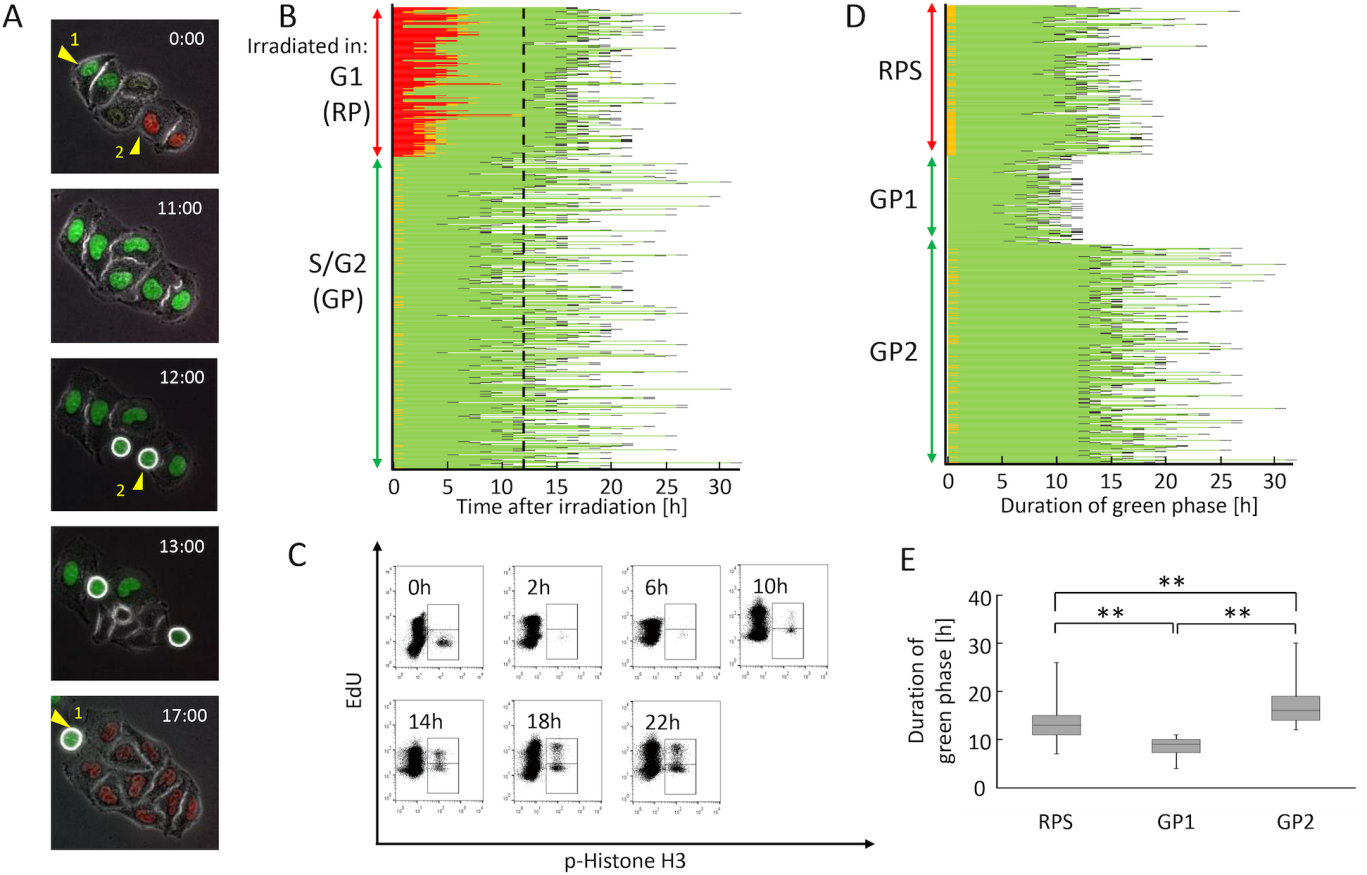
Cell-cycle progression is disrupted after irradiation. (A) A red cell enters M phase faster than a green cell after irradiation. Cell #1, a green cell; Cell #2, a red cell. Round cells represent M phase. Time is shown as hours:minutes. (B) Distribution of red- and green-phase durations in cells irradiated in red or green phase. Black and orange represent M phase and early S phase, respectively. The dotted line indicates the time when the first cell irradiated in red phase reached M phase. (C) Flow-cytometric detection of EdU-labeled mitotic cells after irradiation. Experiments were performed as shown in Fig 1E, except that cells were flash-labeled with EdU immediately after 5 Gy irradiation. (D) Rearrangement of sub-populations in Fig 3B. RPS: G1 population from which the G1-phase duration (red portion) was subtracted; GP1, S/G2 population from the left side of the dotted line; GP2, S/G2 population from the right side of the dotted line. (E) Duration of green phase after irradiation in each sub-population shown in Fig 3D. Data are represented as box-and-whisker plots showing the full range, 25–75% interquartile range (box), and median (bar) from 200 cells in RPS, 138 cells in GP1, and 289 cells in GP2. **, p < 0.01 by Mann–Whitney U test.

We next focused on examining the effects of 5 Gy irradiation on the prolongation of green phase in cells irradiated in red or green phase. During these experiments, we noticed that some cells irradiated in red phase entered M phase faster than cells irradiated in green phase (Fig 3A). To determine whether this was a rare event, we analyzed elongation of green phase until the next M phase in 627 cells irradiated in the red or green phase (Fig 3B). In the figure, M phase cells are depicted as black traces to distinguish them from other green phase cells. The results showed that cells irradiated in green phase could be divided into two cell populations: one that entered M phase more rapidly (GP1) (left fraction of the dashed line) than cells irradiated in red phase (RP); and another (GP2) (right fraction of the dashed line) that entered simultaneously with or slower than RP. Thus, RP caught up with, or in some cases overtook GP2.

To further confirm this phenomenon, we also performed a flow-cytometric analysis, as depicted in Fig 1E (Fig 3C). In this analysis, 5 × 10^4^ cells were used for each time point. Cells were flash-labeled with EdU immediately after irradiation. After apparent G2 arrest, the EdU-negative G2 cells reached M phase (10 h), presumably corresponding to the GP1 fractions described above, and labeled mitotic cells corresponding to GP2 appeared 14 h after labeling. However, the proportion of unlabeled mitotic cells persisted at ~50%, and never went back to 0% (Fig 3C). Considering that the unlabeled G2 fraction was only 8.5% of the total population (Fig 1 and Table 1), and that M phase duration was not influenced by irradiation, the large unlabeled G1 fraction must have entered M phase along with the labeled GP2 fraction. Therefore, we concluded that this event was not rare, but instead occurred frequently after irradiation.

We also performed statistical analysis of the duration of green phase. Specifically, the duration of G1 phase was subtracted from RP, and this difference was designated RPS, and above described GP1 and GP2 were extracted from S/G2 (GP) in Fig 3B. The results are redrawn in Fig 3D. The duration of green phase was significantly shorter in RPS than in GP2 (Fig 3E and S1 Fig). These results clearly demonstrate that elongation of the green phase in RPS was significantly shorter than that in GP2, and that this is the main reason that RP caught up with GP2 after irradiation.

### Prolongation of green phase gradually increases as irradiation timing approaches late G1 phase

**Fig 4:**
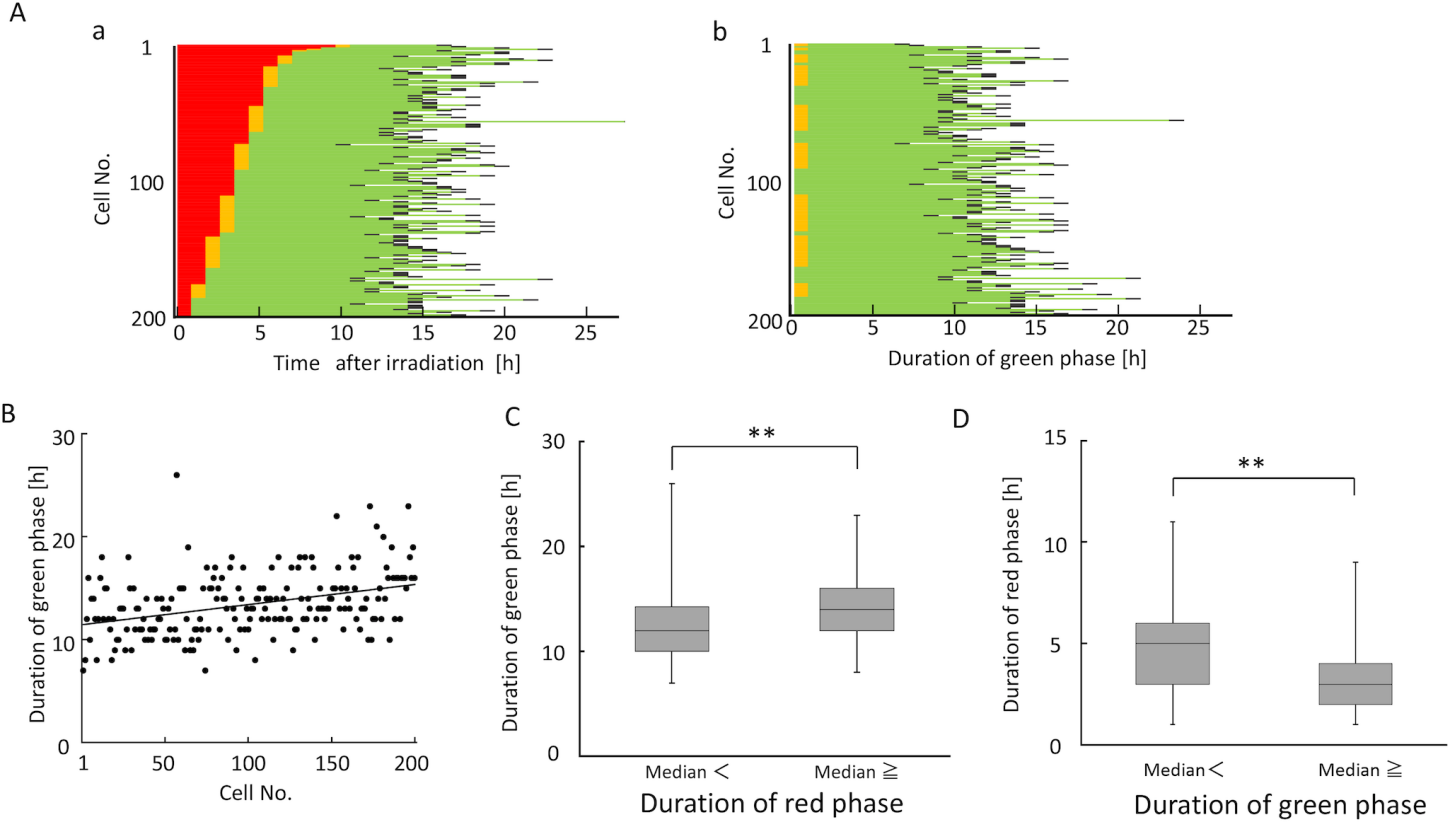
Characteristics of green-phase elongation in cells irradiated in G1 phase. (A) Pedigree analysis in cells irradiated during G1 phase. Rearrangement of cell population irradiated in G1 in Fig 3D, according to the length of red phase (a). Rearrangement of panel a after subtraction of G1-phase duration (b). (B) Quantitative relationship between stage within G1 phase (cell No.) at the time of irradiation and durations of green phase, derived from Fig 4A. Regression line: y = 0.0197x + 11.447, r = 0.372, p < 0.001. (C) Comparison of green-phase duration between cells irradiated in early (red phase duration > 4 h) and late (red phase duration ≤ 4 h) G1 phases, using data from Fig 4A. Data are represented as box-and-whisker plots, as described in Fig 3E. For both populations, n = 100. **, p < 0.01 by Mann–Whitney U test. (D) Comparison of red-phase durations between cells with shorter (≤ 13 h) and longer (> 13 h) green-phase durations after irradiation in G1 phase, using data from Fig 4A. Data are represented as box-and-whisker plots, as described in Fig 3E. For both populations, n = 100. **, p < 0.01 by Mann–Whitney U test.

**Fig 5:**
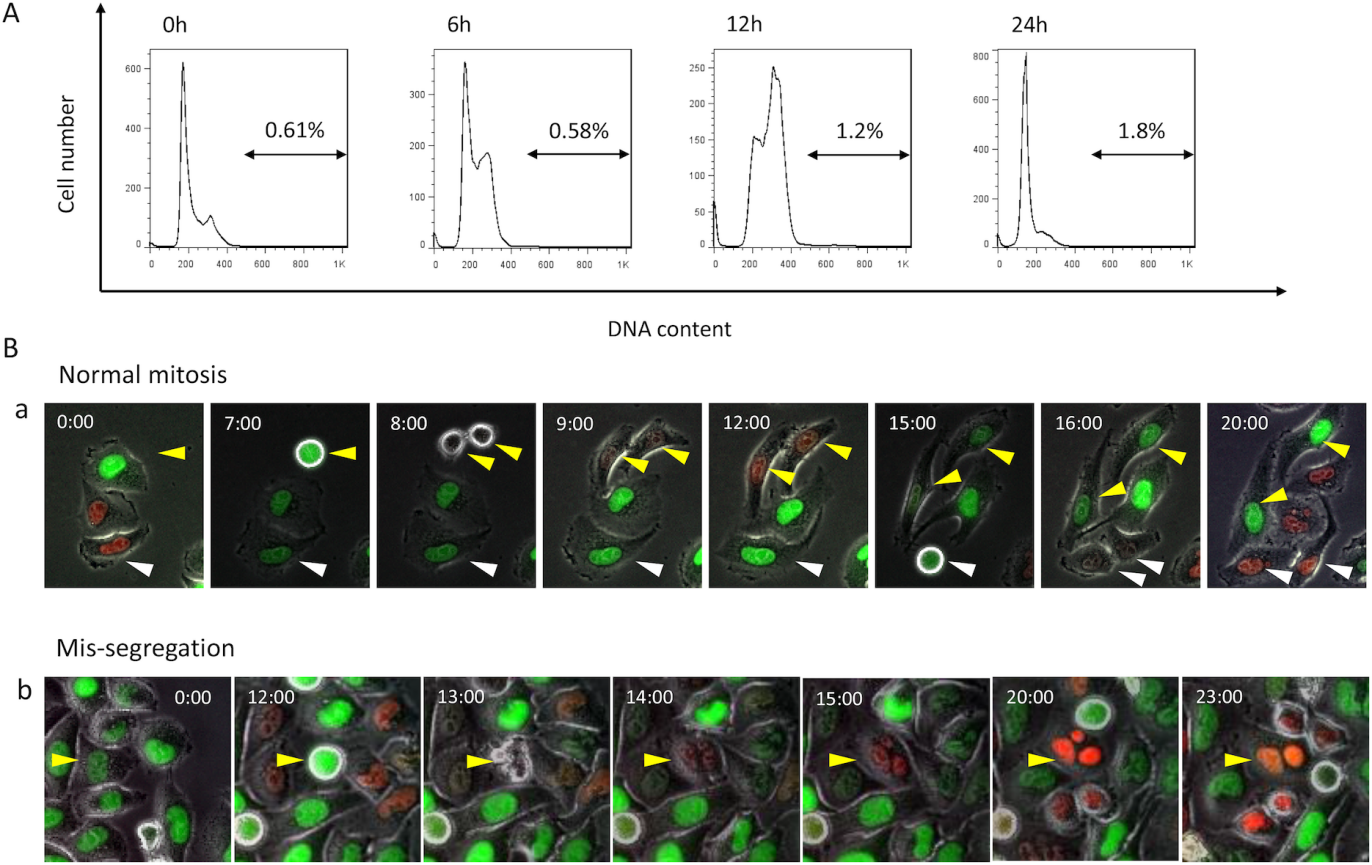
Endoreduplication rarely occurs in HeLa-Fucci cells following 5 Gy irradiation. (A) DNA-content analysis by flow cytometry. Cells were fixed at the indicated times after 5 Gy irradiation, and DNA content was quantitated by flow cytometry. Percentages of polyploid cells are indicated. (B) Detection of normal mitosis and mis-segregation following 5 Gy irradiation. a: normal mitosis. Yellow and white arrowheads represent two examples of normal mitosis. b: mis-segregation. Yellow arrowhead represents an example of mis-segregation. Of more than 600 cells analyzed, only a few cells exhibiting mis-segregation were detected under these conditions. Time is shown as hours:minutes.

Next, we analyzed the effects of irradiation at different stages within G1 phase on the elongation of green phase. Because HeLa cells have non-functional p53, G1 arrest does not occur, and the duration of G1 phase is therefore not affected by irradiation (Fig 2) [25, 26]. Consequently, the duration of red phase, from irradiation to the appearance of green phase, reflected the stage within G1 phase at which a cell was irradiated. RP was sorted according to the length of red phase (Fig 4Aa). Fig 5Ab shows only the duration of green phase after subtraction of the red-phase duration. There was a significant positive correlation between the stage within G1 phase at which a cell was irradiated and the duration of green phase (Fig 4B). Indeed, when G1 phase was divided into two groups (early and late) based on the median value of the red-phase duration (4 h), the green-phase duration was significantly higher in the late-G1 group (Fig 4C and Fig A in S1 File). Conversely, when the green-phase duration was divided into two groups based on the median value (13 h), the group with the longer green-phase duration had a significantly shorter red phase (Fig 4D and Fig B in S1 File). These results strongly support the notion that prolongation of green phase gradually increases as irradiation timing shifts from early to late G1 phase.

### Endoreduplication rarely occurs in HeLa-Fucci cells under the conditions studied

p53-dependent p21 expression after irradiation plays a pivotal role in inhibition of endoreduplication [18]. Because p53 is deficient in HeLa cells, we next investigated whether the phenomena described above were accompanied by endoreduplication. First, we performed DNA-content analysis by flow cytometry and found that the fraction of G2/M cells with 4N DNA content increased gradually, whereas the G1 fraction with 2N content increased and returned to the original distribution up to 24 h after irradiation (Fig 5A). The proportion of polyploid cells was negligible, at least up to 24 h after 5 Gy irradiation (Fig 5A). These results indicated that under the conditions we studied, the majority of cells underwent almost normal mitotic segregation without multiple rounds of DNA replication. Furthermore, the Fucci system was able to identify mis-segregation, which occurs during endoreduplication, as a color change from green to red without cytokinesis [19, 21]. Therefore, the mis-segregated cells were not included in the population with an elongated green phase. Indeed, of more than 600 cells analyzed, we mostly detected normal mitosis with cytokinesis (Fig 5Ba), but identified only a few mis-segregated cells that did not undergo cytokinesis (Fig 5Bb). Taken together, we concluded that endoreduplication rarely occurs under these conditions, even though p53 is non-functional in HeLa cells as a result of HPV infection. Therefore, we reasoned that the elongation of the green phase duration after irradiation simply reflects G2 arrest.

### Establishment of a method for classifying green phase into early/mid/late S and G2 phases

**Fig 6:**
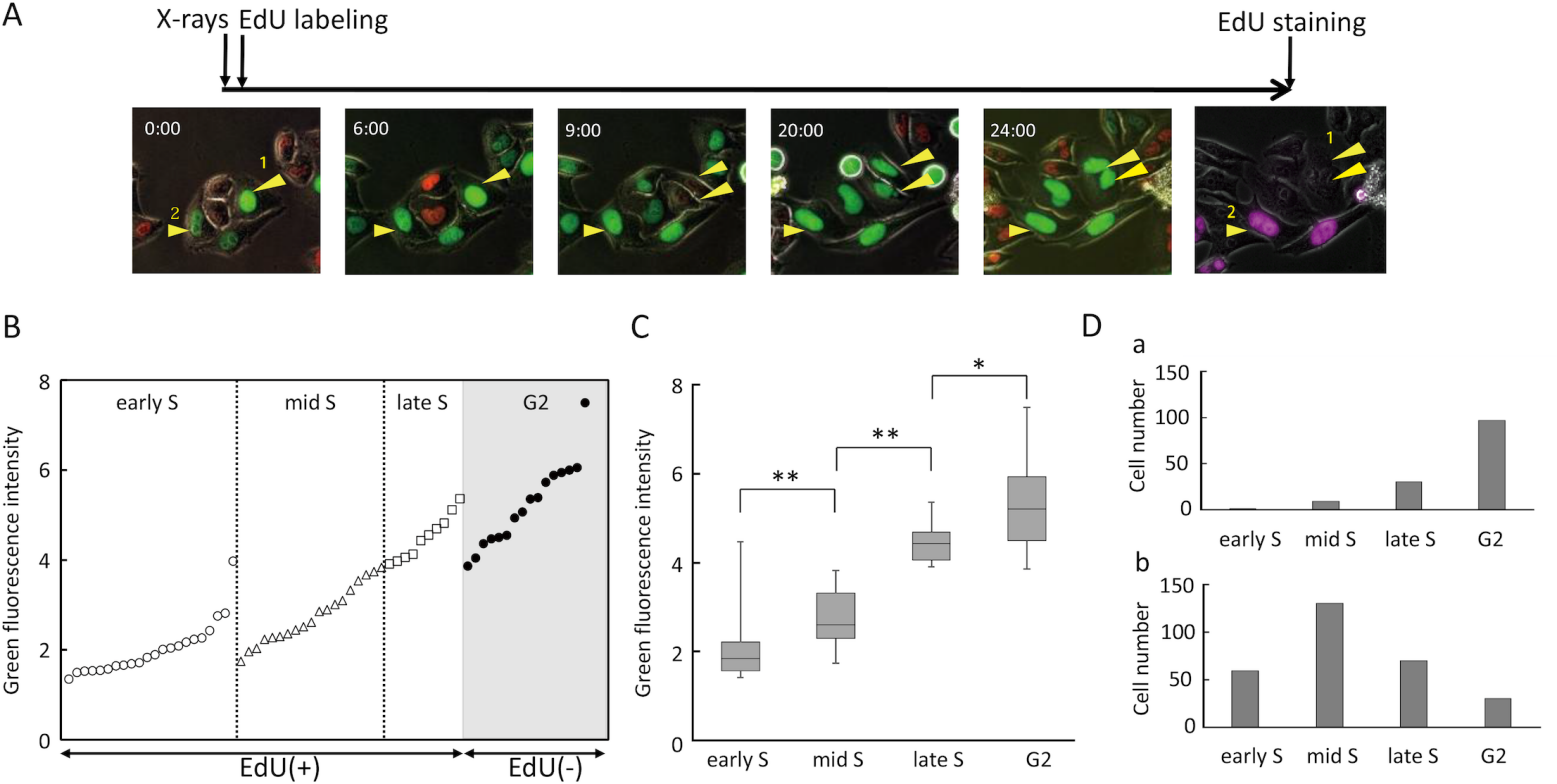
Establishment of a method for classifying green phase into early/mid/late S and G2 phases. (A) Experimental procedure for classification of green phase. Time-lapse imaging was performed for cells flash-labeled with EdU immediately after 5 Gy irradiation, followed by EdU staining. Time is shown as hours:minutes. (B) Classification of green-phase cells into early/mid/late S and G2 phases, according to the method described in A. For simplicity, a subset of cells in each phase is presented. To adjust the fluorescence intensities among different fields, background fluorescence intensity of each image was normalized to 1. (C) Comparison of green fluorescence intensities among sub-groups. Data are represented as box-and-whisker plots, as described in Fig 3E, from 60 cells in early S, 139 cells in mid S, 101 cells in late S, and 127 cells in G2 phase. *, p < 0.05; **, p < 0.01 by Mann–Whitney U test. (D) Validation of the methodology, using GP1 and GP2 from Fig 3D. Cell numbers in each sub-phase within GP1 (a) and GP2 (b) were determined. GP1 and GP2 consisted of 138 and 289 cells, respectively.

In general, the Fucci system is unable to distinguish S and G2 phases [24]. However, we reasoned that such a distinction would be possible if two types of information were simultaneously available: the intensity of green fluorescence, which gradually increases during the green phase due to the constitutively active promoter of Fucci probes [24], and the level of DNA synthesis activity. A simple example of the classification procedure is shown in Fig 6A. At the time of irradiation, a higher intensity of green fluorescence was observed in cell #1 than in cell #2. Immediately after irradiation, EdU was incorporated. After acquisition of time-lapse images, EdU staining was performed, and the results revealed that cell #1 [EdU(−)] and #2 [EdU(+)] were in G2 and S phases, respectively, at the time of irradiation. To apply this method to the 427 previously analyzed green-phase cells (Fig 3B), which had been already EdU-incorporated immediately after irradiation, the following steps were taken to further sub-divide S phase: 1) Green-phase cells were divided into two groups, EdU(+) (S phase) and EdU(−) (G2 phase); 2) The EdU(+) group, emitting both red and green fluorescence, was classified into early S phase, and then sorted according to the quantitative intensity of green fluorescence (ranked from low to high); 3) The remaining EdU(+) group and EdU(−) group were sorted according to the green fluorescence intensity within each group, and cells in EdU(+) group whose intensities overlapped with those in the EdU(−) group (higher intensity) were defined as late S phase; and 4) Finally, the remaining EdU(+) group was defined as mid-S phase. Collective results are shown in Fig 6B; for simplicity, a subset of cells from each sub-S and G2 phase are shown. Among all cells analyzed, the intensity levels differed significantly between each phase (Fig 6C and S2 Fig).

To validate this methodology, we analyzed GP1 and GP2 according to the criteria defined in Fig 3. The results revealed that GP1 and GP2 mainly consisted of cells in G2 and S phases at irradiation, respectively (Fig 6Da and 6Db). This finding was consistent with the results in Fig 3, and we therefore considered the method to be adequately validated.

### Comparison of G2 arrest duration in cells irradiated in G1, early/mid/late S, and G2 phases

**Fig 7:**
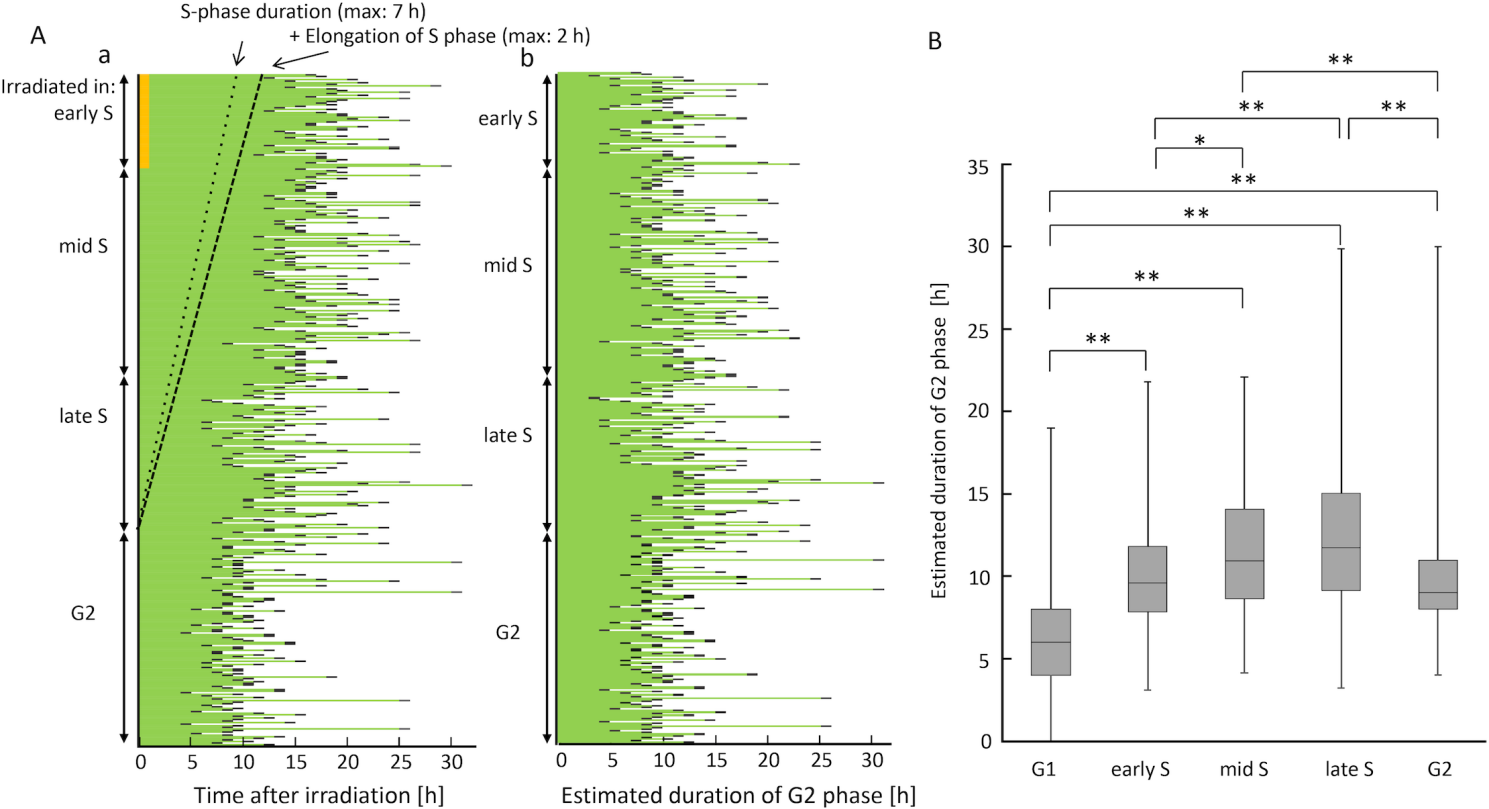
Estimation of G2 arrest durations in cells irradiated in G1, early/mid/late S, and G2 phases. (A) Pedigree analysis of cells irradiated in green phase. a: Distribution of total green-phase durations in cells irradiated in each phase, sorted according to green fluorescence intensities. Two straight lines represent the remaining S-phase durations at irradiation (maximal 7 h for the leftmost cell in early S phase) (lower dashed line) and plus elongation of S phase (maximal 2 h for the leftmost cell in early S phase) (upper dashed line) in each phase. b: Distribution of G2-arrest durations after subtraction of the corresponding S phase and its elongation from the left panel a. (B) Comparisons of G2-arrest durations in cells irradiated in each phase. Data are represented as box-and-whisker plots as shown in Fig 3E. Cell number in each sub-phase is equivalent to that in Fig 5C. *, p < 0.05; **, p < 0.01 by Mann–Whitney U test.

Based on the criteria described above, cells irradiated in green phase were sub-divided into early/mid/late S and G2 phases, and then sorted in the order of green fluorescence intensity within each phase (Fig 7Aa). Using thymidine-blocked early S phase–synchronized cells, we found that S-phase cells exhibited a roughly 2-h delay of S-phase progression (S3 Fig). The results of previous studies indicate that this phenomenon is the result of S-phase checkpoint activation [30]. To obtain the duration of G2 phase including G2 arrest, S-phase elongation in addition to the remaining S-phase duration at irradiation had to be subtracted from the green-phase duration. Two straight lines were assumed to reflect the remaining S-phase duration at irradiation (lower dashed line) plus its elongation (upper dashed line), depending on the positions of S phase at irradiation in Fig 7Aa. Each value on the upper line was then subtracted from each corresponding green-phase duration for cells in S phase at irradiation. The subtracted values were redrawn and are shown in Fig 7Ab. For cells irradiated in G1 phase, S phase was assumed not to be elongated [14]. Finally, each duration in G2 arrest was compared among cells irradiated in G1, early/mid/late S, and G2 phases. G2 arrest for cells irradiated in G1 phase was the shortest among cells irradiated in all examined phases; on the other hand, when cells were irradiated in mid or late S phase, G2 arrest was the longest (Fig 7B and S4 Fig).

### Cell-cycle kinetics observed in irradiated HeLa-Fucci cells are unlikely to occur in p53-functional cells

**Fig 8:**
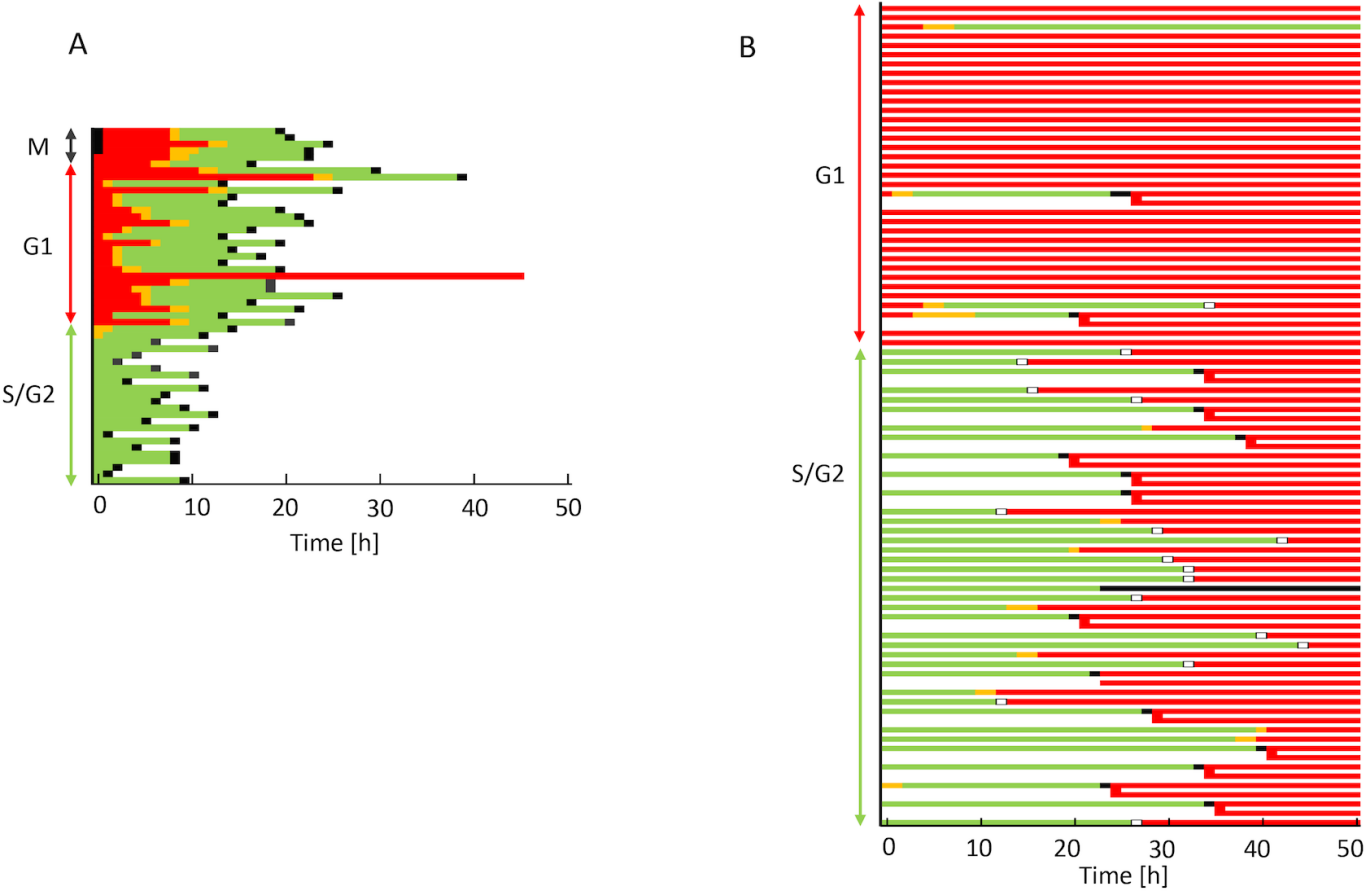
Cell-cycle kinetics in p53-functional cells following irradiation. (A) Order of progression to M phase in red and green cells in non-irradiated BJ1-hTERT-Fucci cells. Mitotic, red, and green cells at the start of observation were monitored until the next entry into M phase. (B) Pedigree analysis on Fucci fluorescence in irradiated BJ1-hTERT-Fucci cells. Time-lapse imaging was performed following 5 Gy irradiation. Each color represents the same as described in Fig 1A and 1D except that the white box represents early G1 phase without any fluorescence after mitotic skipping.

p53 in HeLa cells has been inactivated by human papilloma virus (HPV). Next, we attempted to determine whether cells irradiated in G1 phase catch up with cells irradiated in S phase only when p53 is deficient. For this purpose, p53 was expressed in HeLa-Fucci cells via adenovirus infection. Above multiplicity of infection (MOI) of 60, most cells exhibited apoptosis (data not shown). With MOI < 60, percentages of cells expressing p53 was very low, ~5%, by immunostaining (Fig. A in S2 File). In the latter condition, we observed that a very small number of cells, corresponding to the frequency of p53 expression, exhibited prolongation of red phase and higher red fluorescence (Fig. B in S2 File), which was not observed in uninfected cells. Therefore, we speculated that cells carrying a wild-type p53 gene with Fucci probes may exhibit elongation of red phase, representing G1 arrest. Because appropriate regulation of p53 expression was very difficult in this system, we performed further analysis using BJ1-hTERT-Fucci cells, which were established from h-TERT–immortalized normal human diploid foreskin fibroblasts (BJ-hTERT) with wild-type p53 function [27]. In an unirradiated condition, green cells entered M phase first, whereas red cells entered M phase after almost all the green cells had done so (Fig 8A), like Fucci-HeLa cells (Fig 1D). After 5 Gy irradiation, most cells irradiated in red phase exhibited a remarkable elongation of red phase, and cells irradiated in green phase turned red before the irradiated red cells entered M phase (or underwent mitotic skipping). Thus, the catching up of red cells with green cells, which was characteristic of HeLa-Fucci cells after irradiation, was unlikely to occur in cells carrying a functional p53 gene.

## Discussion

To date, analysis of the effects of irradiation at different cell-cycle phases on subsequent cell kinetics has required isolation of synchronized cell populations by artificial methods. However, this approach poses several potential problems, including 1) incomplete synchronization; 2) redistribution after release from synchronized conditions; 3) side effects of drugs; and 4) failure to reconstruct each response of separately analyzed cell populations after irradiation, assuming that the cells were simultaneously irradiated under asynchronous conditions. Indeed, since the comprehensive study by Terasima and Tolmach using the shake-off method about 50 years ago [14], technical limitations have prevented further substantive progress in this field. The emergence of the Fucci system [24] allowed us, for the first time, to analyze these phenomena in an asynchronous cell populations. Because HeLa-Fucci cells have non-functional p53 and do not exhibit G1 arrest (Fig 2), we expected that observations using this system could focus on elongation of S and G2 phases. The length of the green phase detected using the Fucci system is a useful indicator of cell-cycle kinetics, including G2 arrest, because cells in both S and G2 phases emit green fluorescence. However, given that endoreduplication occurs in p53-deficient cells following DNA damage [17, 18], the situation becomes much more complicated. We found that endoreduplication rarely takes place in HeLa-Fucci cells, at least under the conditions we used (Fig 5); therefore, the duration of the green phase simply reflected G2 arrest. Indeed, HeLa-Fucci cells do not exhibit endoreduplication after etoposide treatment, although mouse mammary epithelial NMuMG cells do [21]. It remains unclear why endoreduplication does not occur in HeLa-Fucci cells, in which p53 is non-functional. However, it is worth noting that we have used a moderate dose of ionizing radiation (5 Gy) whereas previous reports that have studied endopolyploidy have used higher radiation doses [31] or high concentrations of etoposide [21].

In this study, we could simultaneously analyze the effects of irradiation at different cell-cycle phases on individual cell–based kinetics by directly measuring the duration of green phase. Furthermore, it was possible to subdivide S phase and G2 phase by combining information about the green fluorescence intensity properties of the Fucci system and DNA synthesis activity. Following irradiation, most cells irradiated in G2 phase (GP1) entered M phase first, followed by cells irradiated in S phase (GP2) next, although these cells exhibited a remarkably variable elongation of green phase. Interestingly, cells irradiated in G1 phase (RP) entered M phase almost simultaneously with cells in S phase (GP2), and in some cases even earlier. Thus, progression of the cell cycle after irradiation was not strictly determined by the cell-cycle phase at which the cells were irradiated. Because the involvement of endoreduplication was clearly ruled out (Fig 5), we concluded that these observations could be simply attributed to the relationship between the DNA-repair process and the cell-cycle checkpoint.

When repair efficiency of DNA double-strand breaks (DSBs) is reduced, G2 arrest can be elongated, because G2 checkpoint signaling continues to be stimulated [32–35]. Two DSB pathways exist in mammalian cells: non-homologous end joining (NHEJ) and homologous recombination (HR). NHEJ occurs irrespective of cell-cycle phases, whereas HR requires the presence of sister chromatids and therefore occurs only in S and G2 phases [36, 37]; however, it remains unclear how these two pathways are employed during the cell cycle. Karanam et al. recently reported that in G1 phase, DSB repair takes a very short time because only NHEJ is active; on the other hand, in S phase, it takes much longer times for the cell to choose between the two systems [38]. It should be noted that elongation of green-phase duration in cells irradiated in G1 phase was significantly shorter than that in cells irradiated in S phase; consequently, the order of cell-cycle progression was disrupted. The cell kinetics observed in the present study could be explained by the point raised by Karanam et al.: if DSBs are efficiently repaired by NHEJ during G1 phase, G2 arrest should be shortened. Furthermore, it is possible that the duration of green phase gradually increases as irradiation timing approaches late G1 phase because cells enter S phase in the absence of G1 arrest before finishing NHEJ.

Karanam et al. also showed that DNA synthesis activity, rather than the existence of sister chromatids, was well correlated with HR activity, indeed, demonstrating lack of HR in G2 phase [38]. Consistent with this notion, our estimated G2 arrest duration was the largest when cells were irradiated in mid S phase showing the highest DNA synthesis activity; however, the value was also the largest for cells irradiated in late S phase and that was significantly larger for cells irradiated in G2 phase compared to that for cells irradiated in G1 phase. Moreover, the well-established fact that cells are radioresistant in early G1 and late S phases but radiosensitive in early S phase [13, 14] is unlikely to be explained solely on the basis of the choice between the two DSB repair systems. Nevertheless, radioresistance in early G1 phase may be related to our finding that the shortest G2 arrest occurred when cells were irradiated in early G1 phase (Figs 4 and 7), when efficient DSB repair takes place only via the NHEJ pathway. More complicated biological processes may determine the change of radiosensitivity during S and G2 phases. Our method for estimating G2 arrest still required us to make several assumptions in order to determine the remaining S-phase duration at irradiation and elongation. If a fluorescent cell-cycle indicator capable of precisely discriminating S and G2 phases could be developed, more precise information could be obtained.

In conclusion, the Fucci system allowed us to clearly visualize cell-cycle disruption following irradiation in p53-deficient cells in the absence of endoreduplication, which had previously been impossible using synchronized cell populations.

## Supporting Information

S1 Fig Beeswarm plot of Fig 3E. (TIFF)

S2 Fig Beeswarm plot of Fig 6C. (TIFF)

S3 Fig Time course of DNA-synthesizing fraction after release from double–thymidine blocked cells. Early S phase–synchronized cells with or without 5 Gy irradiation were released and flash-labeled with EdU at the indicated times, as described in Materials and Methods. Fractions of labeled cells were determined by flow cytometry.
(TIFF)

S4 Fig Beeswarm plot of Fig 7B. (TIFF)

S1 File Beeswarm plots of Fig 4C (Fig A) and Fig 4D (Fig B). (TIFF)

S2 File Effect of Ad-p53 infection on cell cycle kinetics in HeLa-Fucci cells. Immunostaining for p53 in cells with or without Ad-p53 infection. Cells were infected with or without Ad-p53 (MOI = 20 or 40) and prepared for immunostaining 24 h after virus infection. Nuclei were counterstained with DAPI (Fig A). Fucci fluorescence kinetics after Ad-p53 infection. Cells were infected with Ad-p53 at MOI of 30, and time-lapse imaging was started 16 h after infection. Arrowheads represent cells that exhibited prolonged red phase. Time is shown as hours:minutes after viral infection (Fig B)
(TIFF)
