# Supplementary figures and images for: Effect of X-Irradiation at Different Stages in the Cell Cycle on Individual Cell–Based Kinetics in an Asynchronous Cell Population
Source: PLoS One. 2015 Jun 18;10(6):e0128090. doi: 10.1371/journal.pone.0128090 (PMC4472673; doi:10.1371/journal.pone.0128090)

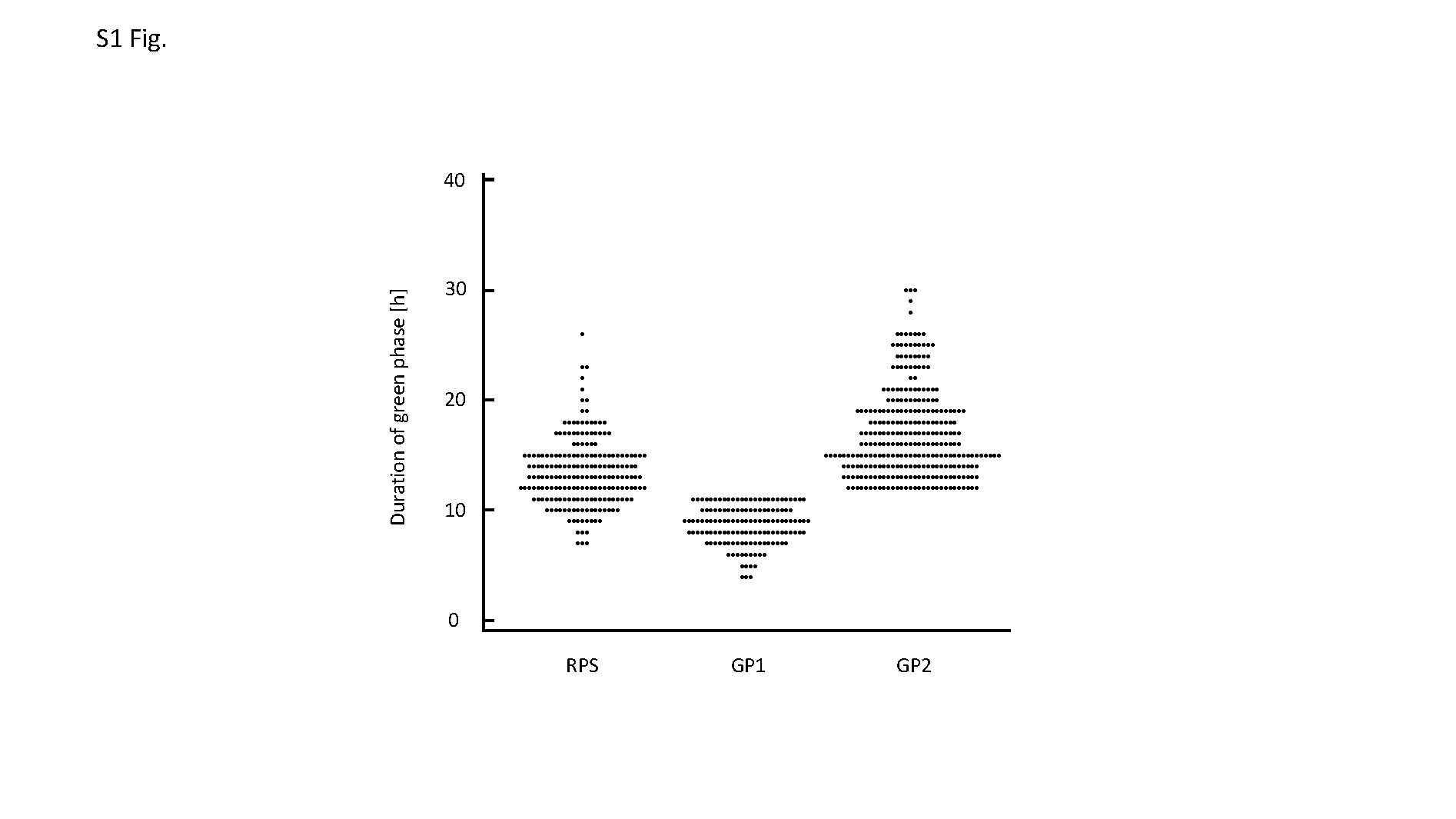

Supplement: S1 Fig — (TIFF) [file pone.0128090.s001.tiff]

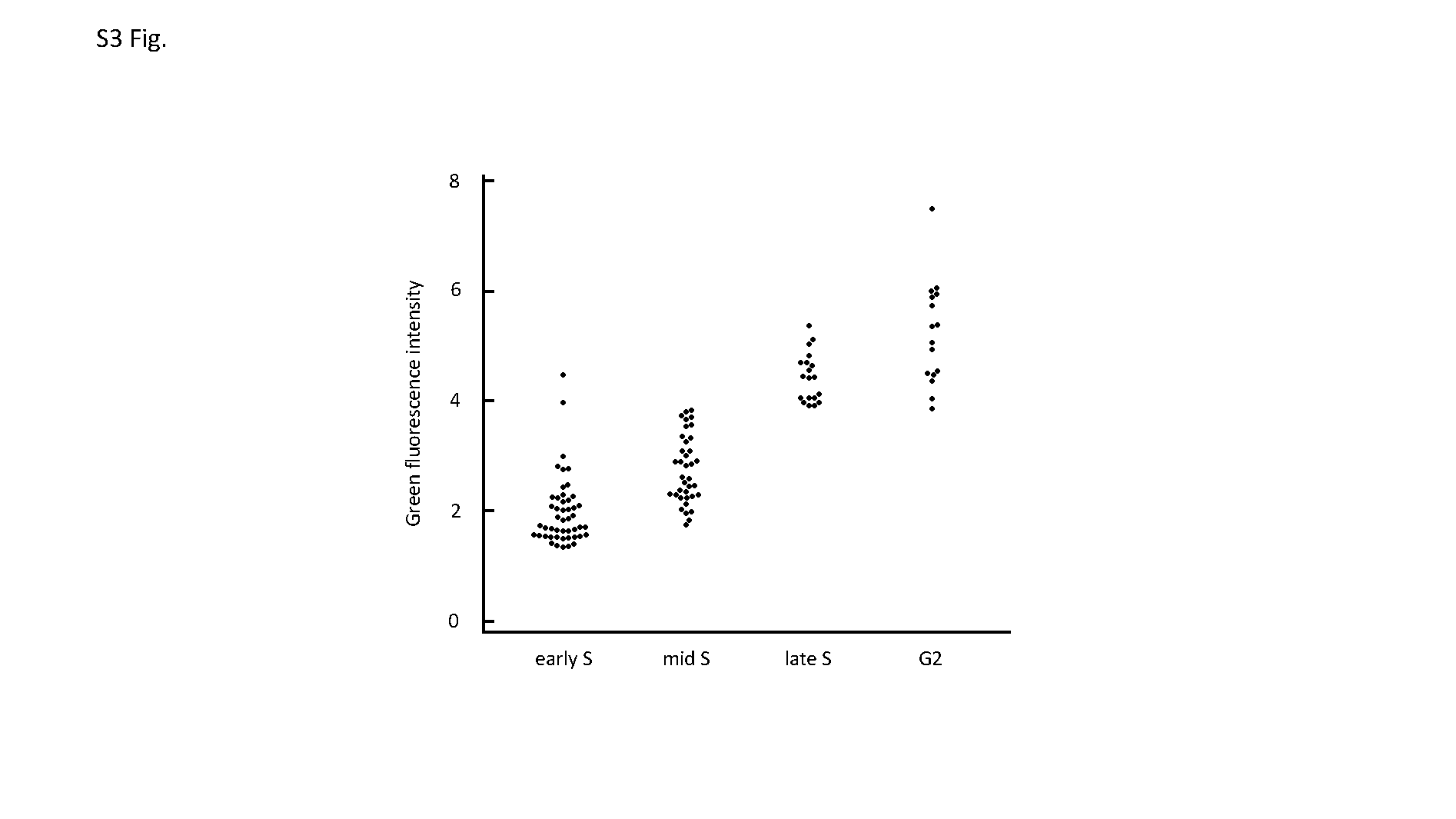

Supplement: S2 Fig — (TIFF) [file pone.0128090.s002.tiff]

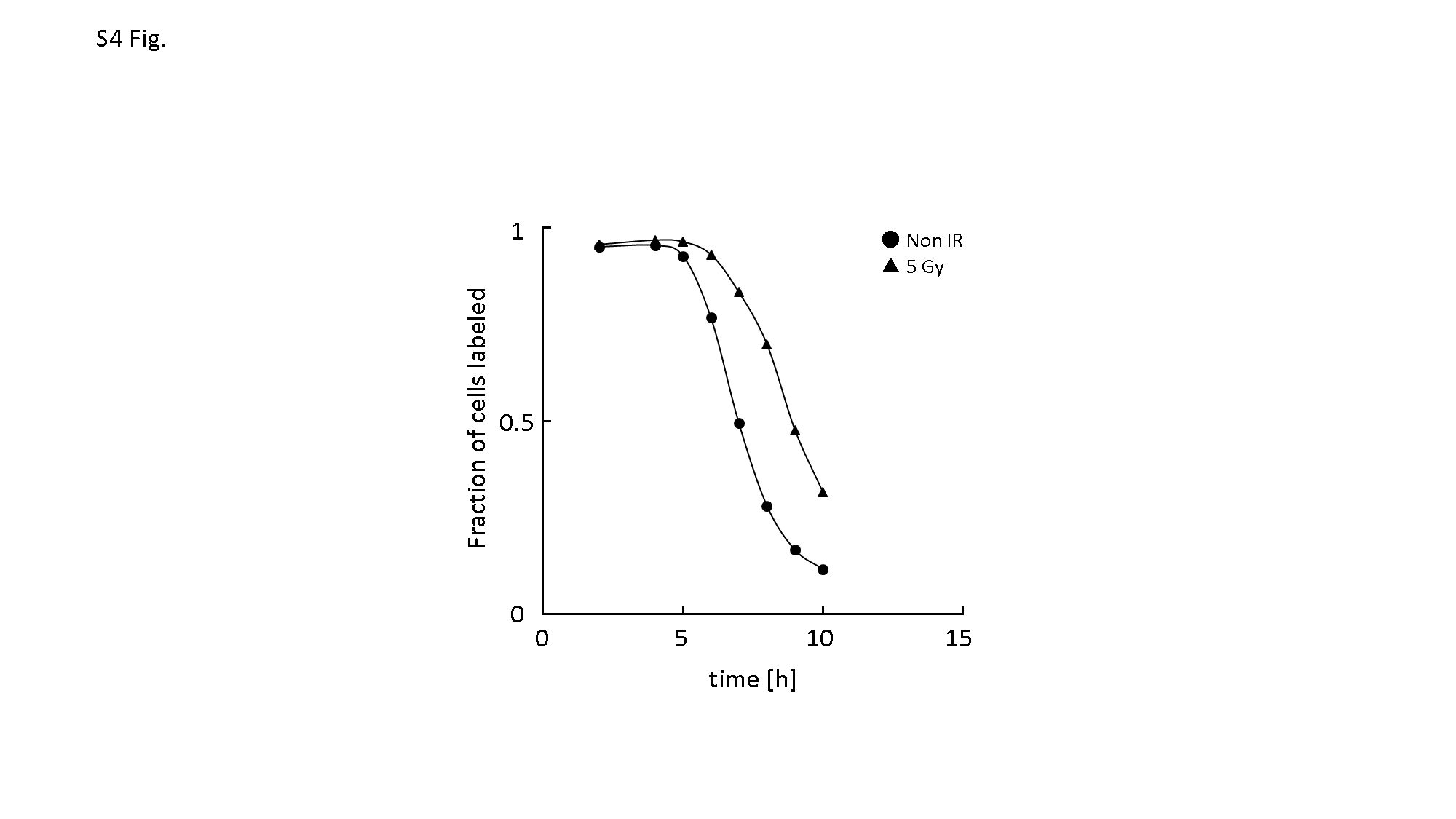

Supplement: S3 Fig — Early S phase–synchronized cells with or without 5 Gy irradiation were released and flash-labeled with EdU at the indicated times, as described in Materials and Methods. Fractions of labeled cells were determined by flow cytometry. (TIFF) [file pone.0128090.s003.tiff]

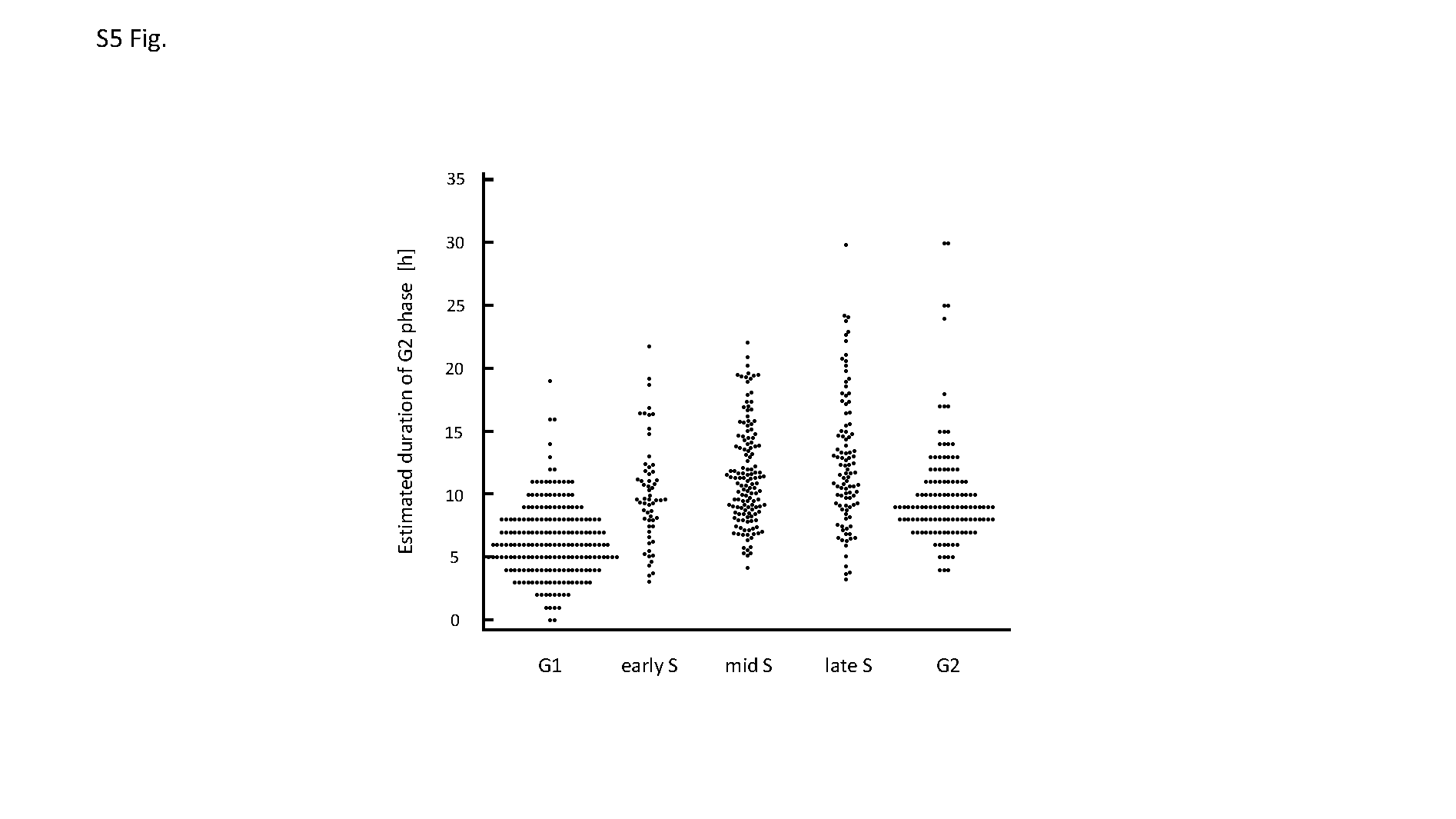

Supplement: S4 Fig — (TIFF) [file pone.0128090.s004.tiff]

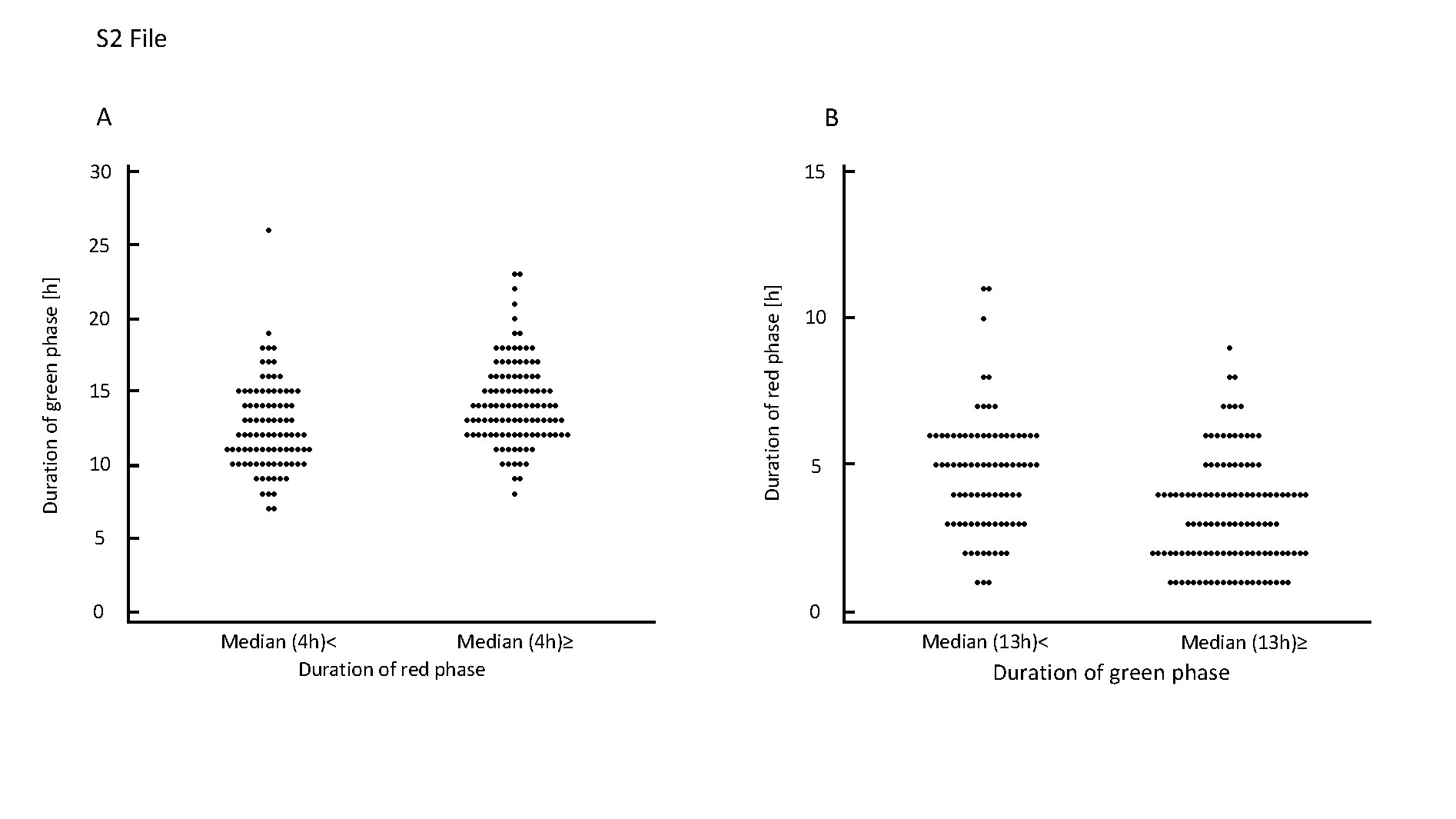

Supplement: S1 File — (TIFF) [file pone.0128090.s005.tiff]

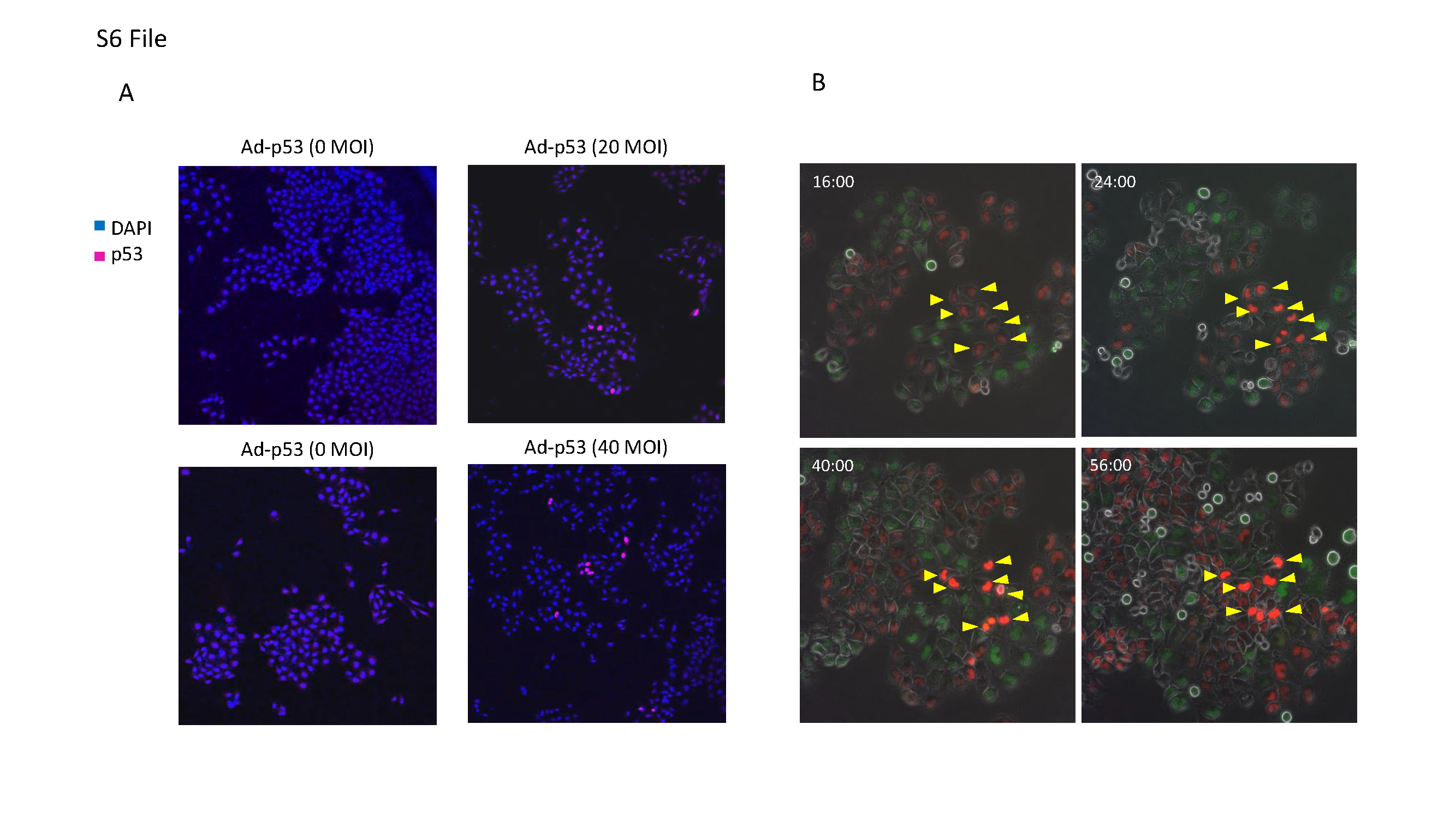

Supplement: S2 File — Cells were infected with or without Ad-p53 (MOI = 20 or 40) and prepared for immunostaining 24 h after virus infection. Nuclei were counterstained with DAPI (Fig A). Fucci fluorescence kinetics after Ad-p53 infection. Cells were infected with Ad-p53 at MOI of 30, and time-lapse imaging was started 16 h after infection. Arrowheads represent cells that exhibited prolonged red phase. Time is shown as hours:minutes after viral infection (Fig B) (TIFF) [file pone.0128090.s006.tiff]
